# Influenza A Infection Stimulates RIG-I and Enhances Effector Function of Primary Human NK Cells

**DOI:** 10.3390/ijms241512220

**Published:** 2023-07-30

**Authors:** Adham Abuelola Mohamed, Sofía Soler, Julia Wegner, Eva Bartok, Sanda Stankovic, Andrew G. Brooks, Martin Schlee

**Affiliations:** 1 Department of Clinical Chemistry and Clinical Pharmacology, University Hospital Bonn, 53127 Bonn, Germany; adham@uni-bonn.de (A.A.M.); julia.wegner@uni-bonn.de (J.W.); 2 Department of Microbiology and Immunology, The Peter Doherty Institute for Infection and Immunity, University of Melbourne, Melbourne, VIC 3000, Australia; sandas@unimelb.edu.au (S.S.); agbrooks@unimelb.edu.au (A.G.B.); 3 Institute of Experimental Haematology and Transfusion Medicine, University Hospital Bonn, 53127 Bonn, Germany; soler@uni-bonn.de (S.S.);; 4 Unit of Experimental Immunology, Department of Biomedical Sciences, Institute of Tropical Medicine, 2000 Antwerp, Belgium; 5 Department of Respiratory Medicine, Alfred Hospital, Melbourne, VIC 3004, Australia

**Keywords:** Influenza A, NK cells, RIG-I, IFN-α/β, innate nucleic acid receptors

## Abstract

Immune surveillance by natural killer (NK) cells and their recruitment to sites of inflammation renders them susceptible to viral infection, potentially modulating their effector function. Here, we analyzed innate RNA receptor signaling in NK cells downstream of direct Influenza A virus (IAV) infection and its impact on NK cell effector function. Infection of NK cells with IAV resulted in the activation of TBK1, NF-ϰB and subsequent type-I IFN secretion. CRISPR-generated knockouts in primary human NK cells revealed that this effect depended on the antiviral cytosolic RNA receptor RIG-I. Transfection of NK cells with synthetic 3p-dsRNA, a strong RIG-I agonist that mimics viral RNA, resulted in a similar phenotype and rendered NK cells resistant to subsequent IAV infection. Strikingly, both IAV infection and 3p-dsRNA transfection enhanced degranulation and cytokine production by NK cells when exposed to target cells. Thus, RIG-I activation in NK cells both supports their cell intrinsic viral defense and enhances their cytotoxic effector function against target cells.

## 1. Introduction

Viral infection can be sensed by germline-encoded nucleic-acid receptors that are expressed in discrete cellular compartments and sense distinct nucleic acid sequences, modifications and/or conformations [1,2,3]. While Toll-like receptors (TLR) 3, 7, 8 and 9 detect nucleic acids in the endosome, RIG-I like helicases (RLH) sense cytosolic RNA, and cGAS detects cytosolic DNA [1,3]. The RLH RIG-I has a preeminent role in the immune recognition of RNA viruses [2,4,5,6]. It is broadly expressed in the cytosol of immune and non-immune cells [3,4] and detects short double-stranded(ds) RNA with 5′ di- or triphosphate termini, as found in genomes and nascent transcripts of RNA viruses [2,4,5,6,7,8,9]. RIG-I activation leads to TBK1/IKKε and nuclear factor kappa B (NF-ϰB) signaling, which, in turn, induces the secretion of antiviral cytokines of the type-I interferon family (type-I IFN), pro-inflammatory and chemotactic cytokines [3,5,10]. Of these, type-I IFN signaling is particularly essential to antiviral defense [11,12]. Canonical type-I IFN signaling via signal transducer and activator of transcription 1 (STAT1) and STAT2 induces a broad antiviral program of interferon-stimulated genes (ISG) devoted to restricting viral replication and spread [1,5,11,12], including nucleic acid receptors, such as RIG-I itself, and direct antiviral effectors, including the interferon-induced protein with tetratricopeptide repeats 1 (IFIT1), which sequesters 5′ triphosphate RNA. In addition to viral RNA, RIG-I can recognize synthetic dsRNA with a 5′ triphosphate (3p-dsRNA) [2,13], and specific RIG-I agonists have been explored as antiviral and antitumor agents [5,14,15,16,17].

Natural killer (NK) cells are cytotoxic innate lymphocytes that also form a key part of the cellular immune surveillance of both transformed and virus-infected cells. NK cells recognize such cells through a repertoire of activating and inhibitory receptors [18,19]. Target cell elimination can occur through cell-to-cell synapse formation and degranulation, transferring of lytic granules of perforin and granzymes, or the release of the FAS-Ligand and TNF-related apoptosis-inducing ligand (TRAIL) [20,21], which activate death receptors and induce extrinsic apoptosis programs. In addition to cytotoxicity, NK cells can influence the immune responsiveness of other cells through the secretion of multiple cytokines and chemokines [18,19,20] including the pleiotropic cytokine TNF and IFN-γ, which promotes antiviral Th1-cell responses, activates antigen presenting cells and induces their MHCII expression [22]. Both NK cell cytotoxicity and cytokine release can be influenced by exposure to cytokines in the (anti-) inflammatory milieu. While type-I IFN is known to pre-activate NK cells, it also upregulates ligands for inhibitory receptors on potential targets (MHCI) and activating receptors on NK cells (e.g., NCR1) [18,19,23].

During viral infection, cell–cell contact during target-cell recognition brings NK cells in close proximity to infected cells that have the potential to directly export mature virions into the NK cells and/or transfer viral entry receptors [24,25]. The negative sense single-stranded RNA viruses Influenza A (IAV) [26,27] and Respiratory syncytial virus (RSV) [28] have shown some capacity to infect primary NK cells. While RSV infection has been reported to enhance NK cell IFN-γ secretion, IAV has been shown to induce apoptosis [29] and in mouse models, reduce NK cell cytokine production and cytotoxicity [27]. Similarly, prolonged exposure of human NK cells to both IAV virions and hemagglutinin has been associated with impaired NK cell effector responses [26]. Although NK cells express high basal levels of RIG-I [30], which could theoretically recognize IAV [2,6] and induce an antiviral response, the role of RIG-I stimulation in IAV modulated NK cell responses has remained unexplored.

In the present study, we aimed to unravel the effect of RIG-I activation triggered by IAV infection or specific, synthetic RIG-I ligands (3p-dsRNA) on NK cell effector function. IAV activated RIG-I in NK cells inducing the activation of the TBK1 and NF-ϰB pathways and type-I IFN secretion. Moreover, IAV also enhanced NK cell effector functions, including degranulation and the secretion of IFN-γ and TNF. Similarly, targeted activation of RIG-I with 3p-dsRNA enhanced IFN release and NK cell effector functions and also protected them from subsequent IAV infection. Altogether, we demonstrate that RIG-I activation by IAV and 3p-dsRNA in NK cells leads to enhanced effector function as well as cytokine release.

## 2. Results

### 2.1. Influenza A Virus Activates NF-ϰB, TBK1 and Type-I IFN Response in NK Cells Via RIG-I

**Figure 1:**
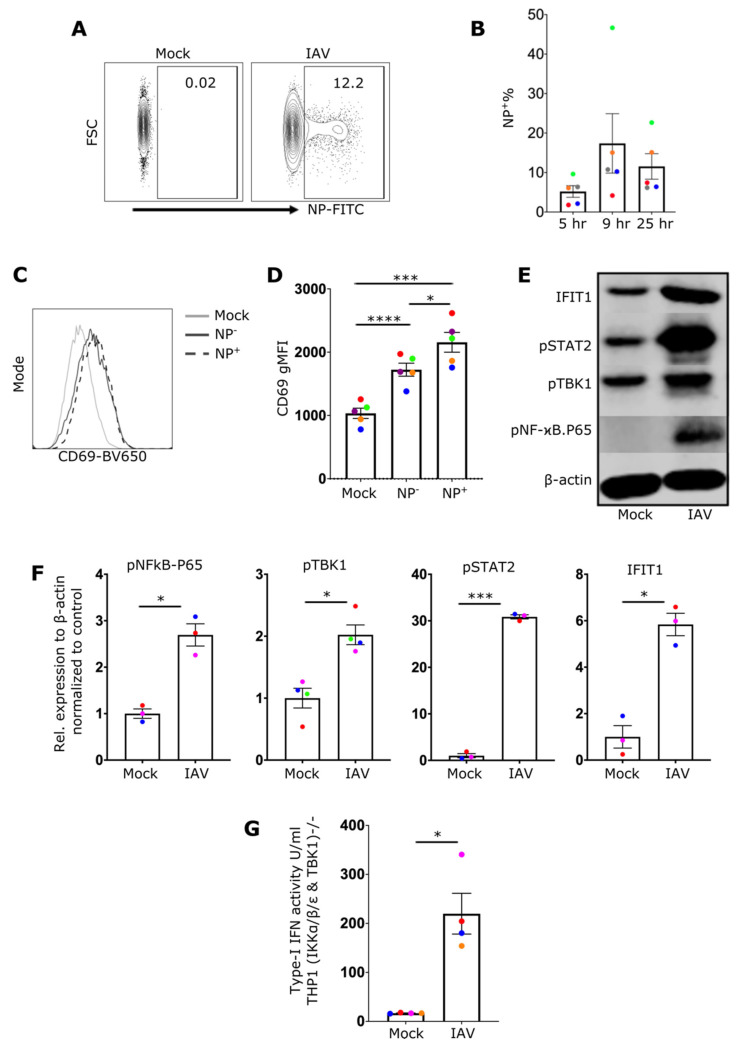
IAV infection of human primary NK cells leads to the activation of NF-ϰB, TBK1 and type-I interferon response. (**A**) Representative NK cell flow cytometry plots 8 h after incubation in media alone (Mock) or 10 MOI influenza A virus (IAV), stained for viral NP to indicate the percentage (%) of infected cells and (**B**) bar graph of % of NP positive cells (5 donors) at 5, 9, and 25 h post-IAV incubation. (**C**) Representative histogram and (**D**) bar graph of CD69 geometric mean fluorescence intensity (gMFI) (5 donors) of mock-treated NK cells, or IAV-exposed NP^-^ and NP^+^ NK cells at 8 h after incubation with IAV. (**E**) Representative NK cell Western blot and (**F**) quantification of Western blot data, after overnight culture in media alone (Mock) or incubation with IAV showing the level of pNF-ϰB.p65, pTBK1, IFIT1 and pSTAT2 (3–4 donors). (**G**) Bar graph showing type-I IFN (IFN-I) activity detected by TBK1^-/-^ IKKα^-/-^ IKKβ^-/-^ & IKKε^-/-^ THP1 dual reporter cells after 24 h incubation with supernatants from NK cells (4 donors). Each symbol represents an individual donor (color is representative of paired samples), and bars show mean ± SEM. Statistical significance was tested using paired t-test for two group comparisons and one-way ANOVA with Dunnett’s correction for more than two groups (* *p* < 0.05, *** *p* < 0.001, and **** *p* < 0.0001).

The ability of IAV to infect primary human NK cells from peripheral blood as well as the subsequent impact of infection on NK cell effector function were evaluated. NK cells were incubated with IAV at a multiplicity of infection (MOI) of 1, 5, or 10, and the proportion of cells expressing viral nucleoprotein (NP) was measured by flow cytometry. At an MOI of 10, the proportion of NP^+^ NK cells was around 18% at 9 h post-infection (Figure 1A). The percentage of NP^+^ cells peaked at 9 h post-infection, but dropped by 25 h, likely reflecting both the proliferation of uninfected cells and death of infected cells (Figure 1B). Interestingly, the viability of infected cells at 25 h post-infection was not significantly different from the mock-treated cells (Suppl. Figure S2A). While there was clear evidence of infected cells at both 9 and 25 h post-infection, the reduction of infectious virus in the supernatant after 24 h showed that the infection remained non-productive (Suppl. Figure S2B).

The next aim was to evaluate IAV-induced NK cell activation by monitoring the expression level of the early activation marker CD69. CD69 was found to be significantly upregulated on the surface of IAV exposed NK cells compared to mock controls. NP^-^ bystander cells expressed more CD69 than mock treated but less than NP^+^ cells suggesting that surface CD69 is induced by intrinsic (e.g., nucleic acid receptors) as well as extrinsic (e.g., type-I IFN) factors (Figure 1C,D). To examine the immune response, NF-ϰB and TBK1 signaling was investigated. Immunoblotting showed that IAV infection induced phosphorylation of both NF-ϰB p65 and TBK1 (Figure 1E,F) and ultimately the production of type-I IFN that was detected in the supernatants of infected NK cells 24 h post-infection (Figure 1G). Type-I IFN can induce autocrine interferon-α/β receptor (IFNAR) activation, leading to STAT1 and STAT2 phosphorylation [11]. STAT2 phosphorylation was detected in infected cells, demonstrating that secreted type-I IFN has an autocrine effect on infected NK cells (Figure 1E,F). In line with the action of secreted type-I IFN, the transcription of the ISG IFIT1 was substantially induced (Figure 1E,F).

**Figure 2:**
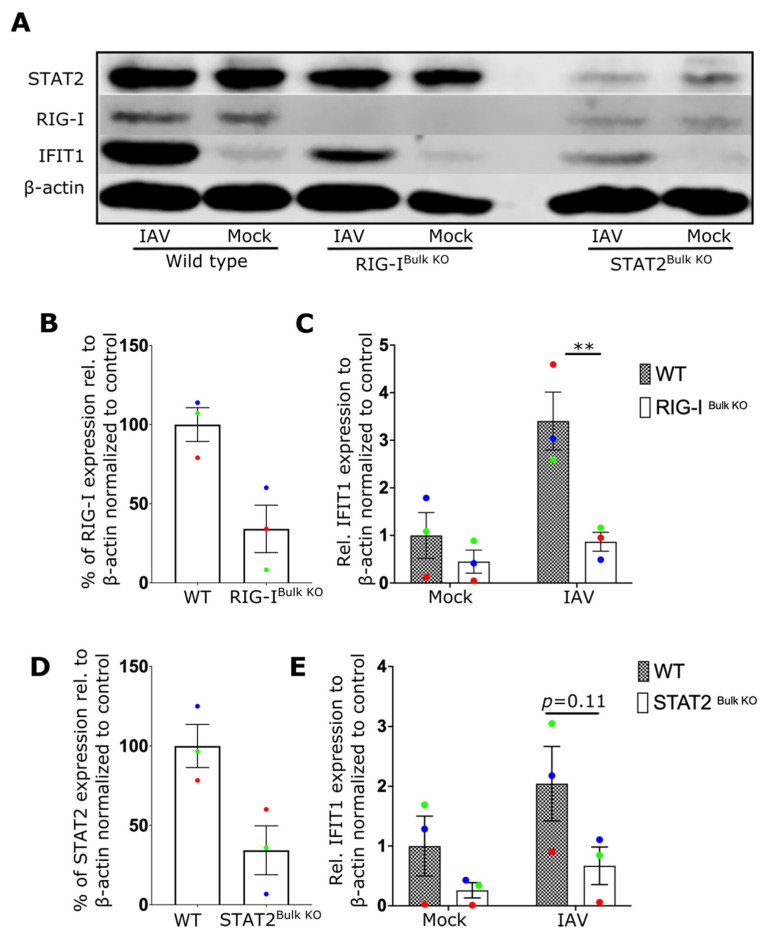
IAV induces an IFN-I response in NK cells via RIG-I and STAT2. (**A**) Representative NK cell Western blot showing expression of IFIT1, RIG-I and STAT2 from wildtype controls (WT), or cells treated to knock out RIG-I (RIG-I^Bulk KO^) or STAT2 (STAT2^Bulk KO^) genes. (**B**) Percentage of RIG-I expression in WT and RIG-I^Bulk KO^ cells. (**C**) Relative expression of IFIT1 in WT and RIG-I^Bulk KO^ cells after overnight incubation with IAV. (**D**) Percentage of STAT2 expression in WT and STAT2^Bulk KO^ cells. (**E**) Relative expression of IFIT1 in WT and STAT2^Bulk KO^ cells after overnight incubation with IAV (3 donors). Each symbol represents an individual donor (color is representative of paired samples). Bars represent mean ± SEM. Statistical significance was tested using two-way ANOVA followed by Bonferroni’s correction for multiple group comparisons (** *p* < 0.01).

We then attempted to determine the innate immune receptor(s) responsible for NF-ϰB and TBK1 activation as well as type-I IFN secretion in NK cells. Although RIG-I is known to sense IAV infection in fibroblasts, epithelial cells and macrophages [6,31,32], no specific studies have identified the critical sensor for IAV in lymphocytes to date. To investigate the role of RIG-I and type-I IFN signaling, RIG-I and STAT2 expressing genes were inactivated in primary human NK cells using CRISPR/Cas9 genome editing. Expression of both proteins was reduced by more than 60% (Figure 2A,B,D) in bulk CRISPR/Cas9 treated cells. IFIT1 induction after IAV expression was dependent on both RIG-I (Figure 2C) and STAT2 (Figure 2E) expression, indicating that RIG-I and STAT2 contribute to the antiviral response of NK cells and that the autocrine action of type-I IFN is a critical component of this response.

### 2.2. IAV Infection Enhances NK Cell Effector Function

**Figure 3:**
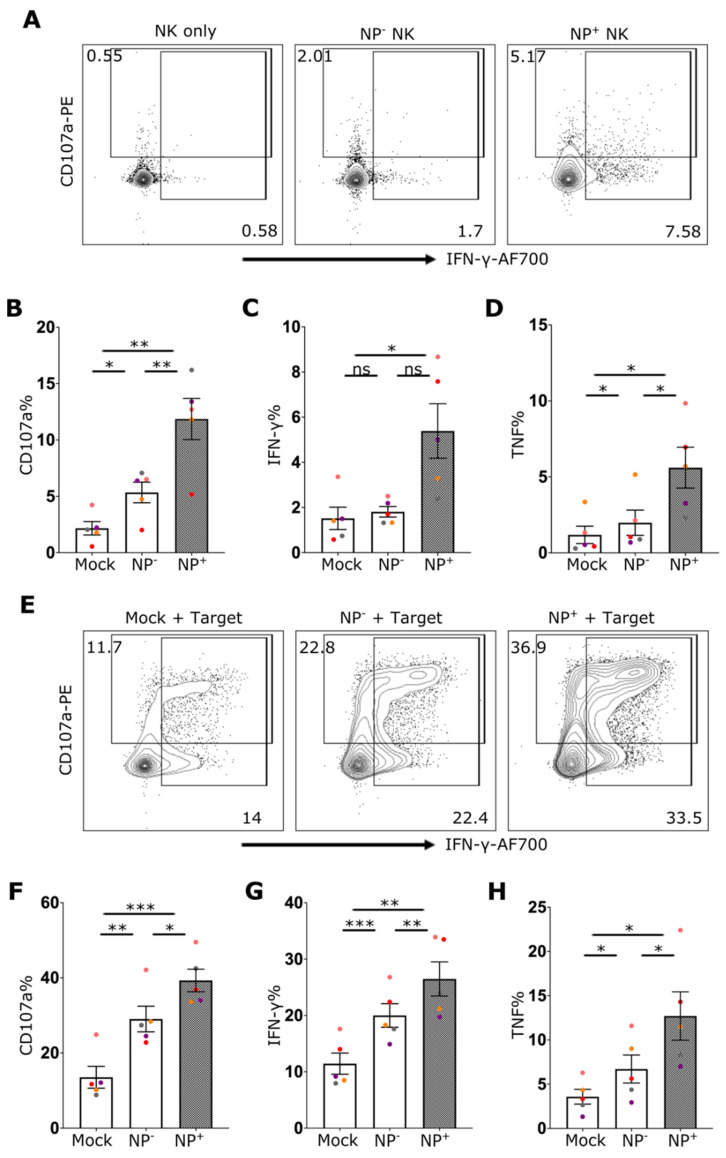
IAV infection of NK cells enhances their effector function. (**A**) Representative flow cytometry plots of NK cells for CD107a and IFN-γ staining from mock culture or NP^−^ (uninfected) and NP^+^ (infected) cells after incubation with IAV. The percentage of cells expressing (**B**) CD107a, (**C**) IFN-γ, and (**D**) TNF is shown (5 donors). (**E**) Representative NK cell flow cytometry plots for CD107a and IFN-γ staining from mock culture or NP^−^ and NP^+^ cells after incubation with IAV in the presence of target cells (721.221) and percentage of cells expressing (**F**) CD107a, (**G**) IFN-γ and (**H**) TNF in the presence of target cells is shown (5 donors). Each symbol represents an individual donor (color is representative of paired samples). Bars show mean ± SEM. Statistical significance was tested using repeated measure one-way ANOVA with Dunnett’s correction (ns = not significant, * *p* < 0.05, ** *p* < 0.01, *** *p* < 0.001).

To investigate the effect of IAV exposure on NK cell function, the capacity of NK cells to respond to the HLA-deficient target cell line 721.221 in the presence or absence of IAV infection was assessed. Even in the absence of target cells, a significant increase in CD107 and IFN-γ could be detected in NP+ cells (Figure 3 A–D). Coculture of NK cells with HLA-class I deficient 721.221 cells induced robust degranulation and cytokine production by NK cells (Figure 3E–H). Here, IAV-exposed NK cells showed robustly increased frequencies of degranulation (CD107a) as well as IFN-γ and TNF cytokine production following stimulation with 721.221 cells (Figure 3E–H). Indeed, the effector and cytokine responses of infected NK cells (NP^+^) was elevated relative to cells that did not stain positive for NP (NP^−^) and mock-treated cells (Figure 3E–H). This suggests an intrinsic role of IAV infection in enhancing NK effector function. Furthermore, following stimulation with PMA/Ionomycin the response of NP^+^ NK cells was again elevated relative to both NP^−^ cells and NK cells that were not exposed to IAV (Suppl. Figure S3).

### 2.3. 3p-dsRNA Activates TBK1 and NF-ϰB Pathways and Induces Type-I IFN in NK Cells

**Figure 4:**
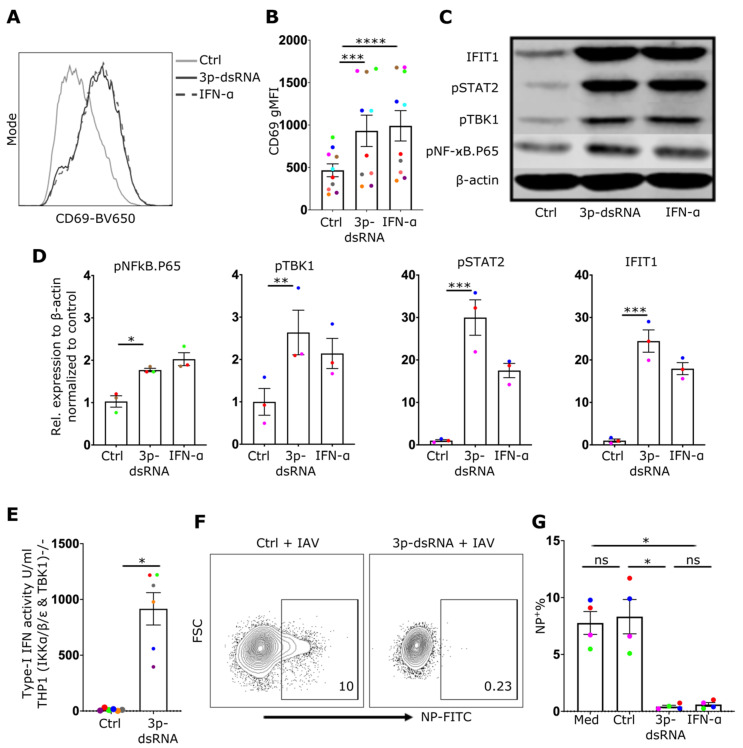
3p-dsRNA activates NF-ϰB, TBK1 and IFN-I response. (**A**) Representative NK cell CD69 gMFI histogram and (**B**) quantification of CD69 gMFI (10 donors), after overnight treatment with 3p-ssRNA (Control, Ctrl), 3p-dsRNA, or IFN-α. (**C**) Western blot from NK cell cultures and (**D**) relative protein expression (3 donors) after treatment of NK cells with 3p-ssRNA (Ctrl), 3p-dsRNA or IFN-α. (**E**) Bar graph showing IFN-I activity of TBK1/IKKα/IKKβ/IKKε-deficient THP1 dual reporter cell line after 24 h incubation with supernatants from NK cells. (**F**) Representative NK cell flow cytometry plots and (**G**) proportion of infected (%NP^+^) cells (4 donors) after pre-treatment with RIG-I ligands (or control) followed by incubation with IAV for 8 h. Med = media only control. Each symbol represents an individual donor (color is representative of paired samples). Bars show mean ± SEM. Statistical significance was tested using paired *t*-test for two group comparison and repeated measures one-way ANOVA with Dunnett’s correction for more than two groups (ns = not significant, * *p* < 0.05, ** *p* < 0.01, *** *p* < 0.001, and **** *p* < 0.0001).

**Figure 5:**
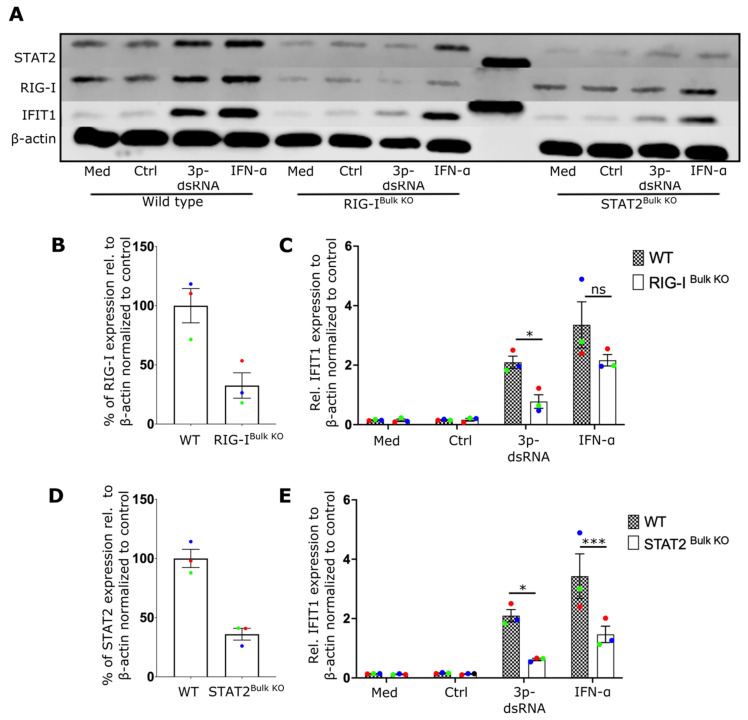
3p-dsRNA induces an IFN-I response in NK cells via RIG-I and STAT2. (**A**) Representative NK cell Western blot for IFIT1, RIG-I, and STAT2 from wildtype (WT), RIG-I^Bulk KO^ and STAT2^Bulk KO^ cells, in the presence of media alone (Med), 3p-ssRNA (Control, Ctrl), 3p-dsRNA or IFN-α. (**B**) Percentage of RIG-I expression in WT and RIG-I^Bulk KO^ NK cells. (**C**) Relative expression of IFIT1 in WT and RIG-I^Bulk KO^ NK cells in the presence of media alone (Med), Ctrl, 3p-dsRNA or IFN. (**D**) Percentage of STAT2 expression in WT and STAT2 ^Bulk KO^ NK cells. (**E**) Relative expression of IFIT1 in WT and STAT2^Bulk KO^ NK cells in the presence of media alone (Med), Ctrl, 3p-dsRNA or IFN (3 donors). Each symbol represents an individual donor (color is representative of paired samples). Bars show mean ± SEM. Statistical analysis was performed using paired t-test for two-group comparisons and two-way ANOVA with Bonferroni’s correction for multiple comparisons (ns = not significant, * *p* < 0.05, *** *p* < 0.001).

Although our data demonstrate that IAV infection activates RIG-I in NK cells, IAV proteins have also been described to alter the NK cell effector response during infection [26,27] and may modulate potential effects of RIG-I signaling in these cells. Thus, to directly assess the impact of RIG-I activation on NK cell effector function, a RIG-I specific synthetic ligand, 3p-dsRNA, or a control RNA that does not activate RIG-I, were delivered into NK cells using lipofection [2,8] in addition to treating NK cells with IFN-α as a positive control. Subsequently, the activation of the RIG-I pathway and the effector functions of NK cells, including degranulation and cytokine production, were evaluated. Delivery of RIG-I ligands in NK cells was confirmed by flow cytometry using FAM-labelled 3p-dsRNA (12% positive cells, Suppl. Figure S4A). Similar to IAV infection, exposure to RIG-I ligands induced upregulation of the activation marker CD69 (Figure 4A,B), phosphorylation of NF-ϰB p65, TBK1, and STAT2 (Figure 4C,D) as well as type-I IFN secretion (Figure 4E). To exclude the contribution of contaminating B cells and monocytes as a source of type-I IFN, a pure population of NK cells (>99%) (CD56^+^, CD3^−^, CD19^−^, CD14^−^) was used to confirm these findings (Suppl. Figure S5A,B). The exposure of cells to type-I IFN has the ability to reduce the susceptibility of many cell types to IAV infection. To investigate whether 3p-dsRNA modulates the susceptibility of NK cells to IAV infection, cells pre-treated with 3p-dsRNA were incubated with IAV. Indeed, compared to controls, NK cells pre-treated with RIG-I ligands or IFN-α exhibited only minimal infection (Figure 4F,G). To confirm autocrine signaling of secreted type-I IFN, neutralizing antibodies were used to block IFNAR2, which resulted in a reduction of STAT2 phosphorylation and IFIT1 protein expression (Suppl. Figure S4B). RIG-I and STAT2 were effectively knocked out, as demonstrated in Figure 5A,B,D. The 3p-dsRNA-induced interferon response was notably diminished in NK cells lacking RIG-I or STAT2 (Figure 5). Efficient knockout of RIG-I (Figure 5B) correlated with the reduced induction of IFIT1 by 3p-dsRNA (Figure 5A,C), while IFIT1 induction by IFNα showed no significant reduction. Conversely, knockout of STAT2 reduced IFIT1 induction by both 3p-dsRNA and IFNα (Figure 5A,E).

### 2.4. RIG-I Ligands Enhance NK Effector Function

**Figure 6:**
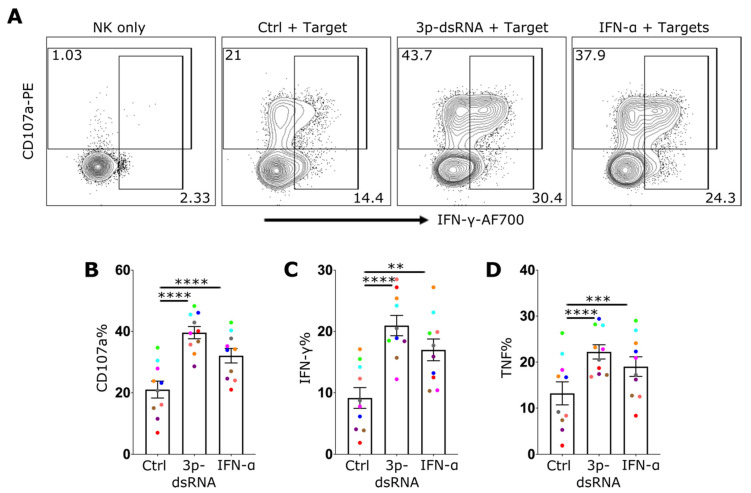
RIG-I stimulation of NK cells enhances their effector function. (**A**) Representative NK cell flow cytometry plot for CD107a and IFN-γ or proportion (%) of NK cells positive for (**B**) CD107a, (**C**) IFN-γ and (**D**) TNF, after incubation in media alone (NK only), or pre-treated with 3p-ssRNA (Control, Ctrl), 3p-dsRNA or IFN-α overnight (followed by incubation with target cells (721.221) for 4 h (10 donors)). Each symbol represents an individual donor (color is representative of paired samples). Bars show mean ± SEM. Statistical analysis was performed using repeated measures one-way ANOVA with Dunnett’s correction (** *p* < 0.01, *** *p* < 0.001, and **** *p* < 0.0001).

While IAV infected NK cells exhibited an enhanced target-cell effector response, it was unclear whether this was directly attributable to the activation of the RIG-I pathway. To test the extent to which this pathway impacts NK cell effector function, NK cells were treated with 3p-dsRNA, IFN-α or control RNA, before incubation with 721.221 target cells. Stimulation with 3p-dsRNA resulted in a significant increase in degranulation (Figure 6A,B) as well as in the production of IFN-γ (Figure 6A,C) and TNF (Figure 6D). This was confirmed with highly pure, sorted NK cells (Suppl. Figure S5). To exclude the indirect activation of NK cells via residual RIG-I ligand stimulation of target cells, THP-1 dual TBK1^-/-^ IKKα ^-/-^ IKKβ ^-/-^ IKKε^-/-^ cells were used as targets. These cells lack RIG-I downstream signaling and therefore do not induce expression of cytokines or antiviral effector proteins. Additionally, the stimulation of these cells with 3p-dsRNA did not lead to enhanced NK cell activation (Suppl. Figure S6A,B). In contrast, incubating them with NK cells transfected with 3p-dsRNA resulted in the same enhancement of NK cell function as observed with 721.221 target cells (Suppl. Figure S6C–E). These findings suggest that the increased activation of NK cells following exposure to 3p-dsRNA is due to intrinsic activation of the RIG-I pathway within NK cells themselves. In summary, 3p-dsRNA significantly enhanced NK cell effector functions in an RIG-I dependent mechanism.

## 3. Discussion

RIG-I is an essential detector of RNA virus infection in many tissues and cell types and is crucial for an effective antiviral immune response [3]. While RIG-I activation with synthetic RNA has been shown to enhance NK cell-mediated killing of melanoma cells [30,33], exposure of NK cells to IAV, a RIG-I stimulating virus, has been reported to reduce NK cell cytotoxicity [29,34]. However, the potential contribution of RIG-I signaling to the NK cell response to IAV infection remains poorly understood. By investigating RIG-I activation and downstream signaling after RIG-I ligand or IAV exposure and using CRISPR/Cas9 mediated genome editing in primary NK cells, we systematically investigated the effects of RIG-I activation during IAV infection. Here, we demonstrated that both IAV infection and stimulation with a synthetic RNA ligand of RIG-I induced CD69 expression, TBK1 phosphorylation and type-I IFN secretion in a RIG-I dependent manner. Moreover, upon recognition of target cells, IAV infection and RIG-I stimulation also enhanced NK cell degranulation (CD107a surface expression) and IFN-γ as well as TNF production. Of note, our flow cytometric analyses showed that infected (NP positive) cells had higher levels of activation than non-infected bystander (NP negative) cells, indicating a strong direct stimulus generated by IAV in the infected cell.

IFN-α incubation could mimic the effects of both IAV and the RIG-I ligand on NK effector functions, and both deletion of STAT2 from NK cells and antibody-mediated neutralization of IFNAR prevented the induction of the type-I IFN response. Altogether, these data indicate that IAV/RIG-I-mediated effects on NK cell function required the type-I IFN signaling pathway.

A previous study observed the transfection of synthetic RIG-I ligand into NK cells, but not pre-incubation with IFN-β enhanced killing of melanoma cells in vitro [30]. Thus, in certain tumor types, activation of RIG-I might induce additional genes over and above those induced by type I IFNs, which we did not observe here. However, using myeloid tumor cells (THP1) or HLA-I-deficient B-lymphoblastoid cells (721.221) as target cells, we observed a similar enhancement of IFN-γ, TNF or CD107a expression induced by IAV, 3p-dsRNA or pre-incubation with IFN-α. Interestingly, in contrast to 3p-dsRNA and IFN-α, exposure to IAV was able to substantially induce IFN-γ production even if target cells were absent. This might reflect the capacity of the IAV-HA on the surface of virions to crosslink activating receptors such as NKp46 suggesting not only that IAV enhances NK effector function via RIG-I/type-I IFN, but that NK cells need additional NK cell receptor stimulation for functional activation (reviewed in Luczo et al. [35]).

While our data contrast with previous studies that show impairments of NK cell cytotoxic activity by IAV infection or exposure to IAV [34,36], Lin et al. [33] demonstrated, in accordance with our data, that exposure to IAV induced the expression of CD69 and enhanced degranulation as evidenced by increased CD107a surface expression on human NK cells. However, although the study showed enhanced activation and degranulation, the authors observed reduced target cell killing, which may be attributable to apoptosis of the infected NK cells leading to reduced effector to target cell ratios [29]. In contrast to Lin et al. [29], we observed little NK cell death 24 h after virus infection, which might be due to the different virus strains used: RG-PR8-Brazil78 HA, NA (H1N1) in our study and WSN virus/33 (H1N1) in their study. It is conceivable that the NS1 of different strains differ in their capacity to suppress RIG-I activation.

Regardless, our data show clear RIG-I-dependent activation of virus-infected NK cells and that RIG-I activation both enhances NK cell effector function and contributes to a more effective antiviral response. Activation of RIG-I in NK cells may therefore enhance their capacity to target virus infected cells that themselves are neither able to secrete type-I IFN nor to recruit antigen presenting cells that can produce type-I IFN. Cells that are already infected and produce viral particles also express viral suppressors of IFN induction such as NS1, a potent inhibitor of RIG-I activation, which prevent cytokine production. When newly recruited to the site of infection, NK cells could conceivably directly produce type-I and type-II IFN after sensing the IAV genome from released viral particles. Nevertheless, the in vivo relevance of such a scenario remains to be elucidated. On the other hand, since recruited NK cells are exposed to high concentrations of virus at the site of infection, a functional RIG-I pathway that induces a strong autonomous antiviral response in NK cells might be crucial to minimizing the risk of uptake, replication and spread of the virus in the body. Thus, strong RIG-I signaling in NK cells support both an enhanced cellular effector function against infected target cells as well as the cell autonomous antiviral defense of the NK cell itself.

## 4. Materials and Methods

### 4.1. Primary Cells, Cell Lines, Tissue Culture

Human peripheral blood (PBMCs) mononuclear cells were obtained by density gradient centrifugation from buffy coats of healthy donors after written informed consent and institutional review board approval. NK cells were isolated from PBMCs using negative selection with EasySep™ Human NK Cell Isolation Kit (Stemcell Technologies, Cologne, Germany, #17955) (Suppl. Figure S1) and cultured at 37 °C in a humidified atmosphere of 5% CO_2_ in NK MACS^®^ Medium (Miltenyi Biotec, Bergisch Gladbach, Germany, #130-114-429) containing 10% of fetal bovine serum (Thermo Fisher Scientific, Langerwehe, Germany, #10437028), 100 U/mL penicillin, 100 μg/mL streptomycin (Thermo Fisher Scientific, #15140122), and 100 U/mL human IL-2 (Miltenyi Biotec, Bergisch Gladbach, Germany, #130-097-743) overnight before performing experiments. Human immortalized (EBV transformed) HLA negative B-lymphoblastoid cells (721.221 cells) were grown at 37 °C in humidified atmosphere of 5% CO_2_ in RPMI 1640 medium supplemented with 10% fetal bovine serum, 100 U/mL penicillin, and 100 μg/mL streptomycin. THP1 dual cells (InvivoGen, California, USA) were cultured in the same medium and conditions used for 721.221 cell line. They express a secreted luciferase reporter gene under the control of an ISG54 minimal promoter in conjunction with five IFN-stimulated response elements. To generate THP1 dual (TBK1^-/-^ IKKα ^-/-^ IKKβ ^-/-^ IKKε^-/-^) the corresponding genes were inactivated by CRISPR/Cas9 mediated gene editing as described [37]. In those cells, signaling downstream of RIG-I is abrogated while signaling downstream of the IFNAR is still intact. 

### 4.2. IAV Infection and RIG-I Stimulation of NK Cells

NK cells were infected with a reassortant Influenza A virus (IAV) strain from PR8 and A/Brazil/11/1978: RG-PR8-Brazil78 HA, NA (H1N1) at 10 multiplicities of infection (10 MOI) in serum-free medium, then washed 2X after 1 hr with PBS and incubated for different durations as described in each experiment. RIG-I ligands were generated by in vitro transcription with a Transcript Aid T7 in vitro transcription kit (Thermo Fisher Scientific, Langerwehe, Germany, #K0441) using annealed DNA oligonucleotides as a dsDNA template (sequence: TTGTAATACGACTCACTATAGGGACGCTGACCCAGAAGATCTACTAGAAATAGTAGATCTTCTGGGTCAGCGTCCC) as described before [38]. The negative control for RIG-I-like receptor activation, a single stranded 3p-RNA, was generated using the dsDNA template with the sequence CGCGCGTAATACGACTCACTATAGGGAGCGCAGACGCGAGCGCGGCACGGCCGCCAAGGCGAGAC. Lipofectamine 2000 (Thermo Fisher Scientific, Langerwehe, Germany, #11668019) was used as transfection agent. Then, 5 µg/mL RNA and 2.5 µL/mL lipofectamine were complexed and added to 10^6^ NK cells/mL for the stimulation. Where indicated, cells were stimulated with recombinant IFN-α2a (1000 U/mL) (Miltenyi Biotec, Bergisch Gladbach, Germany, #130-108-984).

### 4.3. Western Blot

Cells (at least 4 × 10^5^) were centrifuged at 500× *g* for 5 min and washed with PBS after harvesting, then lysed with 1× Laemmli buffer containing PhosStop (Roche, Basel, Switzerland, #4906837001) and protease inhibitor (Roche, Basel, Switzerland, #4693116001), vortexed and incubated at 95 °C for 5–7 min in a thermomixer, shaking at 600 rpm, to denature the proteins. After that, equal volumes (equivalent to 4 × 10^5^ cells/run) were loaded on a 10% SDS-PAGE gel for electrophoresis until the desired separation was achieved. Then, proteins were transferred to nitrocellulose membranes, and proteins were stained individually with primary antibodies anti-β-actin mouse mAb (LI-COR Bioscience, Bad Homburg, Germany, #926-42212), anti-IFIT1 rabbit mAb (Cell Signaling, Frankfurt, Germany, #14769), anti-phospho-p65 rabbit mAb (Cell Signaling, Frankfurt, Germany, #3033), anti-phospho-TBK1 rabbit mAb (Cell Signaling, Frankfurt, Germany, #5483), anti-RIG-I rabbit mAb (Cell Signaling, Frankfurt, Germany, #3743), anti-STAT2 rabbit mAb (Cell Signaling, Frankfurt, Germany, #72604), or anti-phospho-STAT2 rabbit mAb (Cell Signaling, Frankfurt, Germany, #88410). After staining, proteins were detected using the Odyssey Imaging system (LI-COR Biosciences, Bad Homburg, Germany). The relative expression levels of the target protein were quantified using Image Studio Lite software by normalizing the signal intensity of the target protein to the signal intensity of β-actin. Then, different conditions were normalized to controls.

### 4.4. Flow Cytometry and Degranulation Assay

Cells were washed with flow cytometry wash buffer (2% of FBS, 0.5 µM EDTA in PBS) and then stained for 30 min at 4 °C with anti-hCD3 APC-Cy7 (BD Bioscience, Heidelberg, Germany, #557832), anti-hCD56 APC (Miltenyi Biotec, Bergisch Gladbach, Germany, #130-113-310) or FITC (BD Bioscience, Heidelberg, Germany, #562794), anti-hCD69-BV650 (BD Bioscience, Heidelberg, Germany, #563835), or anti-hCD107a-PE (BD Bioscience, Heidelberg, Germany, #555801) antibody. Antibodies were diluted 1:200 in flow cytometry wash buffer. Cell viability was assessed using Fixable Viability Dye eFluor™ 780 at a dilution of 1:1000 (Thermo Fisher Scientific, Langerwehe, Germany, #65-0865-14). For degranulation assays, 50,000-100,000 NK cells were cocultured with 721.221 cells as target cells in a 1:1 ratio for 4 h at 37 °C. Golgi Stop (BD Bioscience, Heidelberg, Germany, #554724) and Golgi Plug (BD Bioscience, Heidelberg, Germany, #555029) were added to the media along with CD107a-PE antibodies at a dilution of 1:200 during the whole assay time. For intracellular protein staining, cells were fixed and permeabilized using eBioscience™ Foxp3/Transcription Factor Staining Buffer Set (Thermo Fisher Scientific, Langerwehe, Germany, #00-5523-00) and then incubated with anti-hIFN-γ-AF700 (BD Bioscience, Heidelberg, Germany, #557995), anti-hTNF-PECy7 (BD Bioscience, Heidelberg, Germany, #560678) and anti-NP-FITC (Abcam, Berlin, Germany, #ab210526) for 30 min at 4 °C. Again, antibodies were diluted 1:100 in permealization buffer. Attune NxT (Thermo Fisher Scientific, Massachusetts, USA) and LSR Fortessa (BD Biosciences, Heidelberg, Germany) machines were utilized for sample acquisition and data analysis was performed using FlowJo™ (v9) software (Oregon, USA). Differential gating for NK cell and target cells was accomplished via size difference (FSC/SSC) and the expression of CD56. NK cells were defined as viable, single, and CD3^−^ CD56^+^ cells. The purity was quantified to be >92% for each donor (Suppl. Figure S1). Gates of NP+ cells were drawn as uninfected cells were not expressing the viral protein, gating of CD107a, IFNg and TNF was based on unstimulated cells. CD69 gMFI was used instead of gating since CD69 expression increases showing a shift in the whole population. The sample analysis was performed on Attune NxT and LSR Fortessa flow cytometers. The technical quality control was generally performed daily for the Attune and weekly on the Fortessa flow cytometers. In all experiments, however, the unstimulated cells were also used as negative controls that acted as technical references for experiments. We have addressed this in the methods section.

### 4.5. IFN-I Reporter Assay

To determine the type-I IFN reporter activity, cell-free supernatants were collected 16-20 h after infection or stimulation, 100 μL supernatant were added to medium-free THP1-dual TBK1^-/-^ IKKα ^-/-^ IKKβ ^-/-^ IKKε^-/-^ cells and incubated for 24 h. Then, 30 μL from the supernatants were mixed with 30µL coelenterazine (1 μg/mL in water) in a white 96-well F-bottom plate and luciferase activity was measured immediately using an EnVision 2104 Multilabel Reader device (PerkinElmer, Massachusetts, USA).

### 4.6. CRISPR-Editing of Primary NK Cells

We used pre-designed crRNAs to edit genes in primary human NK cells using CRISPR/Cas9 system (Integrated DNA Technologies, Leuven, Belgium). Two crRNAs targeted the RIG-I gene (*DDX58*) on the negative strand (GGATTATATCCGGAAGACCC) and positive strand (GATCAGAAATGATATCGGTT), respectively. Additionally, we used one crRNA targeting STAT2 on the positive strand (AAGTACTGTCGAATGTCCAC). As a negative control, a pre-designed non-targeting crRNA (Intergrated DNA Technologies, Leuven, Belgium, #1072544) was used in parallel to the genes of interest (referred to as “Wild type” in the results). Electroporation was performed using the P3 Primary Cell 4D-Nucleofector X Kit S (Lonza, Cologne, Germany, #V4XP-3032) and the 4D Nucleofector system (Lonza, Cologne, Germany) with the CM137 program. Up to 1.5 × 10^6^ primary human NK cells per reaction were used to ensure maximum viability and delivery of the CRISPR/Cas9 mixture. For each reaction, we prepared the CRISPR/Cas9 mixture by combining 2 µL of 100 µM crRNA for each crRNA, equal amounts of tracrRNA (Integrated DNA Technologies, Leuven, Belgium, #1072534), 1.7 µL of Cas9 enzyme (Integrated DNA Technologies, Leuven, Belgium, #1081058), and 1 µL of 100 µM enhancer (Integrated DNA Technologies, Leuven, Belgium, #1075916). The mixture was completed to 25 µL per reaction with electroporation buffer included in the P3 Primary Cell 4D-Nucleofector X Kit S (16.65 µL P3 media to 3.65 µL supplement). After electroporation, cells were incubated for 3 days before stimulation and infection were performed.

### 4.7. Statistical Analysis

Statistical calculations were performed using GraphPad Prism 9 (California, USA). Each donor is represented by a single dot in all figures, with different colors distinguishing between donors. The data shown is derived from at least three independent experiments, each using a total of three or more donors, as indicated in each figure legend. The bars indicate the mean ± standard error of the mean (SEM) across all donors. Paired t-tests were used to determine the significance of differences between two groups, while one-way ANOVA for repeated measures followed by Dunnett’s correction were used for more than two groups. For multiple comparisons, we used two-way ANOVA followed by Bonferroni’s correction. Statistical significance is indicated by asterisks as follows: * *p* < 0.05, ** *p* < 0.01, *** *p* < 0.001, and **** *p* < 0.0001.

## Data Availability

Not applicable.

## Supplementary Materials

The following supporting information can be downloaded at: https://www.mdpi.com/article/10.3390/ijms241512220/s1.
