# Supplementary material for: Influenza A Infection Stimulates RIG-I and Enhances Effector Function of Primary Human NK Cells
Source: Int J Mol Sci. 2023 Jul 30;24(15):12220. doi: 10.3390/ijms241512220 (PMC10419028; doi:10.3390/ijms241512220)
Supplement: Supplementary file 1 [file ijms-24-12220-s001.zip › ijms-2493750-supplementary.pdf]

Supplementary Material

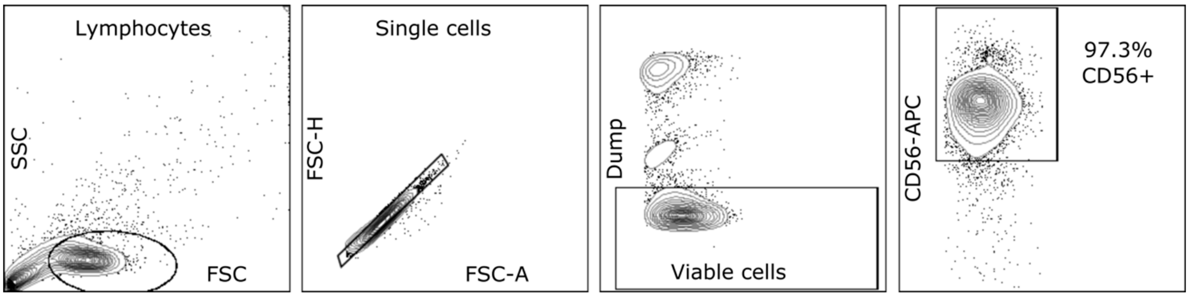

Supplementary Figure S1. Gating strategy and NK purity post-enrichment.
Representative flow cytometry plots of PBMCs. The lymphocytes were gated using the forward and side scatter plot and by excluding doublets, dead cells and $CD3^+$ cells. The NK cells were identified as $CD56^+$ $CD3^-$ cells.

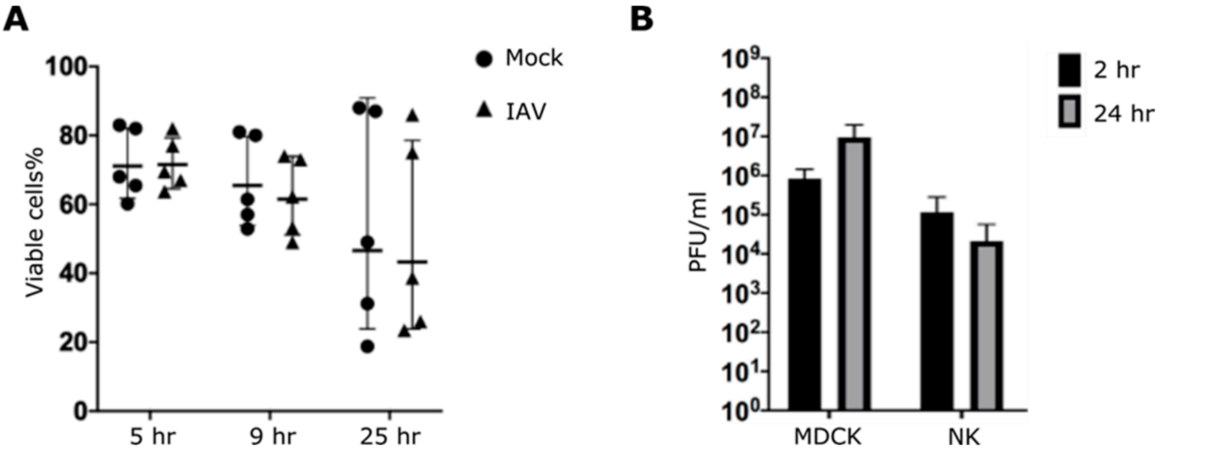

Supplementary Figure S2. Cell viability and live virus in the supernatant after incubation with IAV

A) Graph represents the viability of mock-treated and IAV-infected NK cells (10 MOI) at 5, 9, and 25 hrs post infection Each symbol represents a unique sample. Data are shown as mean± SD, n=5. **B)** Bar graph of viral titer (PFU/ml), measured by plaque assay, from cell-free supernatants of NK cells and MDCK cell cultures at 2- and 24-hrs post-incubation with IAV. Bars show mean± SEM, n=3.

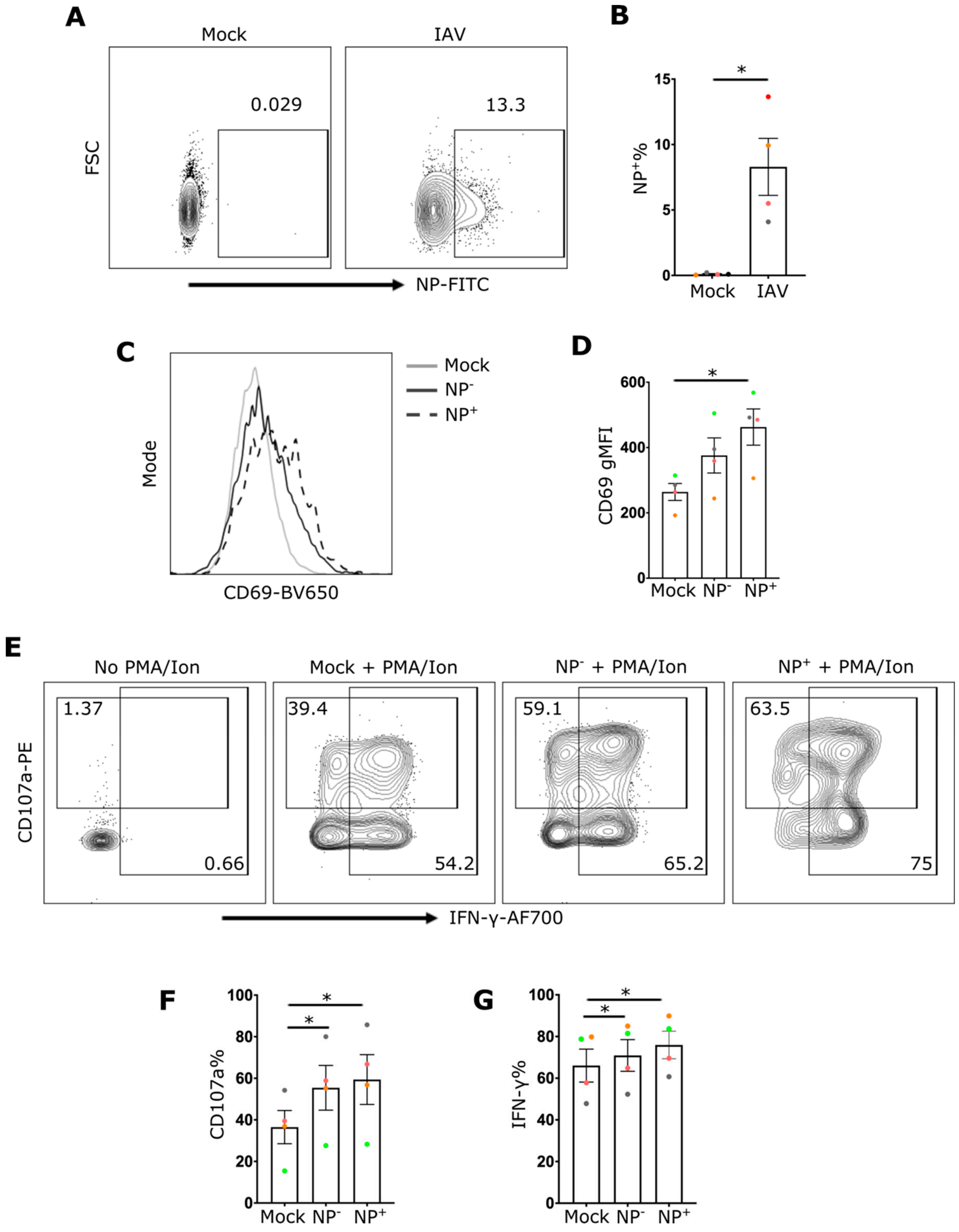

Supplementary Figure S3. IAV infection of NK cells enhances their activation by PMA/Ionomycin

A) Representative NK cell flow cytometry plots and **B)** quantification of NP^+ NK cells at 8 hrs after media alone incubation (Mock) or incubation with IAV (n=5). **C)** Representative histogram of CD69 gMFI for mock-treated, NP^- and NP^+ NK cells and **D)** quantification of

CD69 gMFI (n=4). **E)** Representative NK cell flow cytometry plots from media alone (Mock), NP^- and NP^+ NK cells stimulated with PMA/Ionomycin and **F)** quantification of $CD107a^+$ cells and G) IFN-γ^+ cells from these cultures. Each symbol represents an individual donor (color is representative of paired samples). Bars show mean ± SEM. Statistical analysis was performed using paired t-test (*$p < 0.05$, **$p < 0.01$).

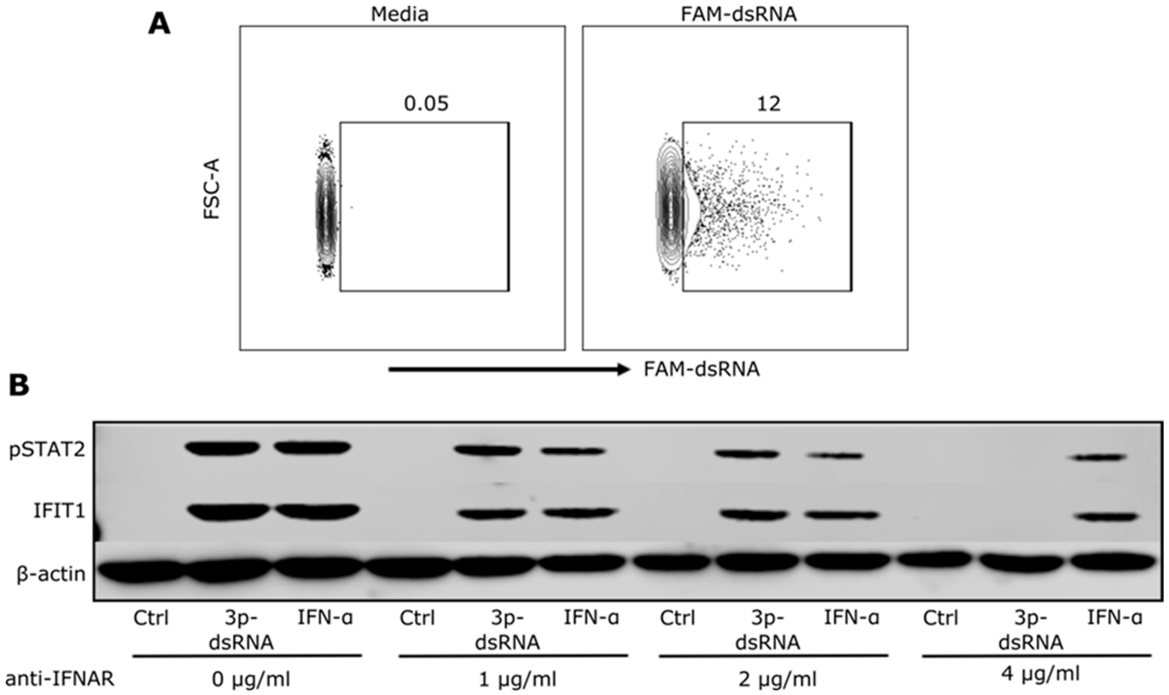

Supplementary Figure S4. 3p-dsRNA induces IFN-I secretion and stimulates IFNAR-dependent IFIT1 induction

A) NK cell flow cytometry plots after overnight incubation in media alone or after treatment with FAM-labelled dsRNA. **B)** Western blot of NK cells for phosphorylated STAT2 (pSTAT2) and IFIT1 proteins without, or pre-treated with anti-IFNARα antibodies (1, 2, or 4 μg/ml) for 1 hr before overnight incubation with media alone, 3pdsRNA, or IFN-α.

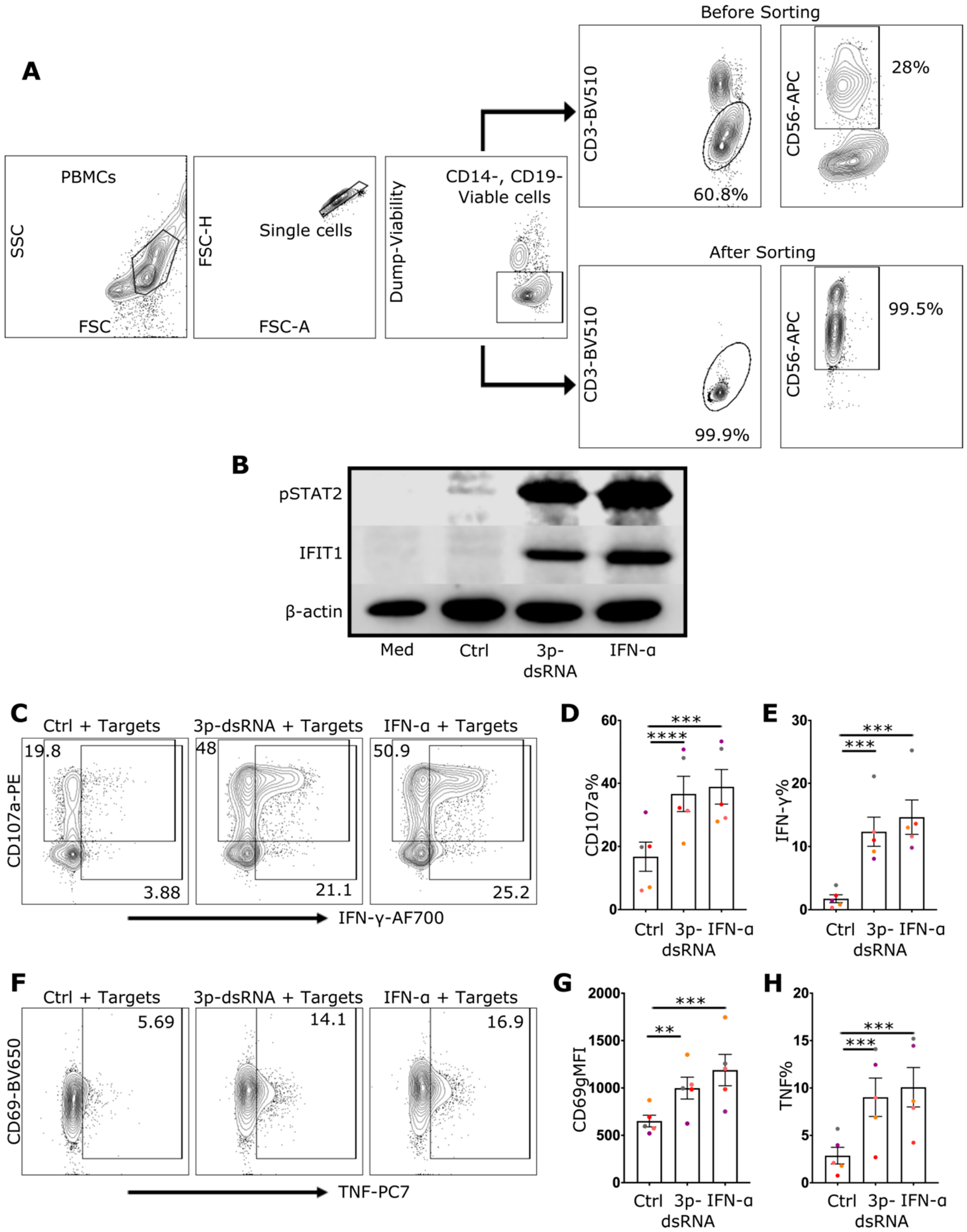

Supplementary Figure S5. Sorted NK cells reproduce results from cultures obtained from NK negative selection kits

A) Representative flow cytometry plots showing the gating strategy for cell sorting and the purity of NK cells before and after sorting. **B)** Western blot from sorted NK cells treated as labelled **C)** Representative sorted NK cell flow cytometry plots showing CD107 and IFN-γ staining, **D)** the percentage (%) of CD107a- or **E)** IFN-γ-positive cells (n=5), in the presence of 3p-ssRNA (Ctrl), 3p-dsRNA or IFN-α. Each symbol represents an individual donor (color is representative of paired samples). Bars show mean ± SEM. Statistical analysis was

performed using repeated measures one-way ANOVA with Dunnett's correction (*$p < 0.05$, **$p < 0.01$, ***$p < 0.001$, and ****$p < 0.0001$).

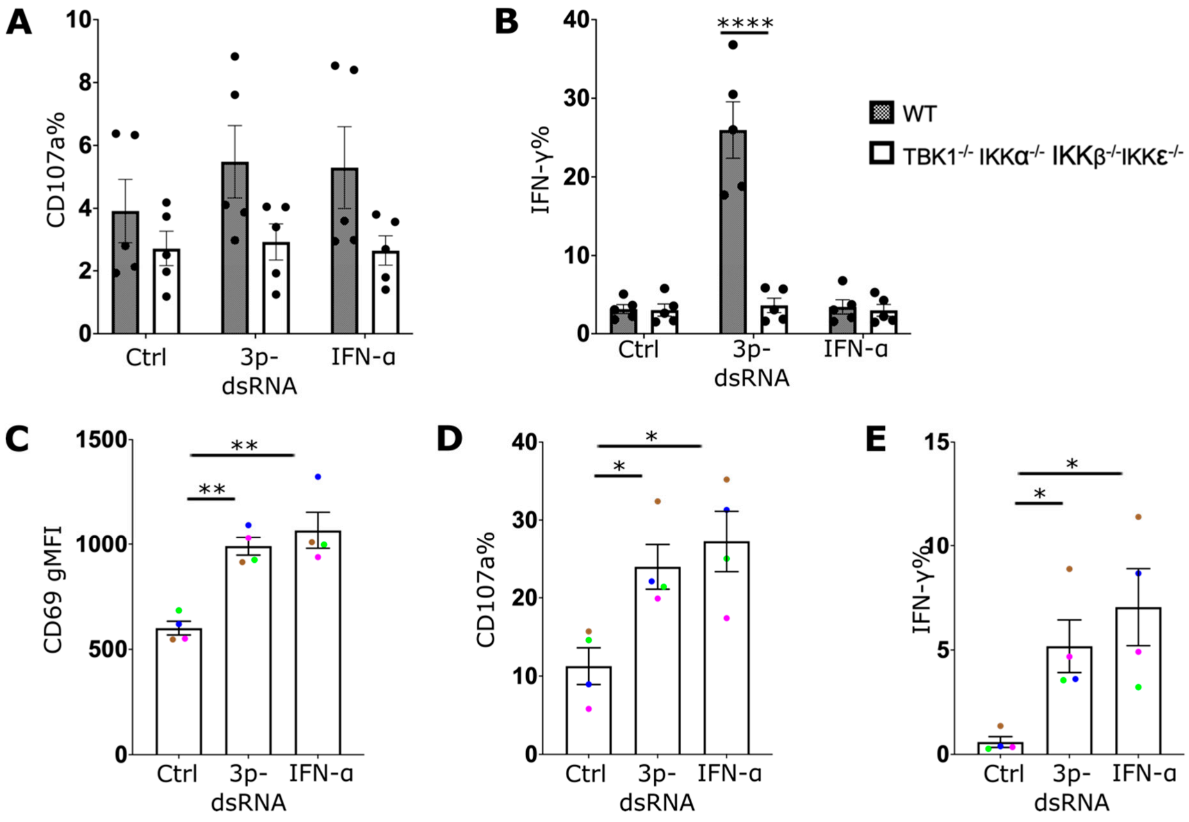

Supplementary Figure S6. RIG-I stimulation enhances NK cell function towards TBK1$^{-/-}$ IKKα$^{-/-}$ IKKβ$^{-/-}$IKKε$^{-/-}$ THP1 targets

A) The percentage (%) of CD107a and **B)** IFN-γ positive NK cells pre-incubated (for 4 hrs) with 3p-ssRNA (Ctrl), 3p-dsRNA or IFN-α followed by co-culture with WT THP1 cells or TBK1$^{-/-}$ IKKα$^{-/-}$ IKKβ$^{-/-}$ IKKε$^{-/-}$ THP1 cells (n=5). **C)** The CD69 gMFI, **D)** % CD107a and **E)** % IFN-γ positive NK cells pre-incubated overnight with 3p-ssRNA (Ctrl), 3p-dsRNA or IFN-α, followed by co-culturing with TBK1$^{-/-}$ IKKα$^{-/-}$ IKKβ$^{-/-}$ IKKε$^{-/-}$ THP1 cells (n=4). Each symbol represents an individual donor (color is representative of paired samples). Bars show mean ± SEM. Statistical analysis was performed using two-way ANOVA with Bonferroni's correction for multiple comparisons and repeated measures one-way ANOVA with Dunnett's correction for more than two groups (*$p < 0.05$, **$p < 0.01$, ***$p < 0.001$, and ****$p < 0.0001$).

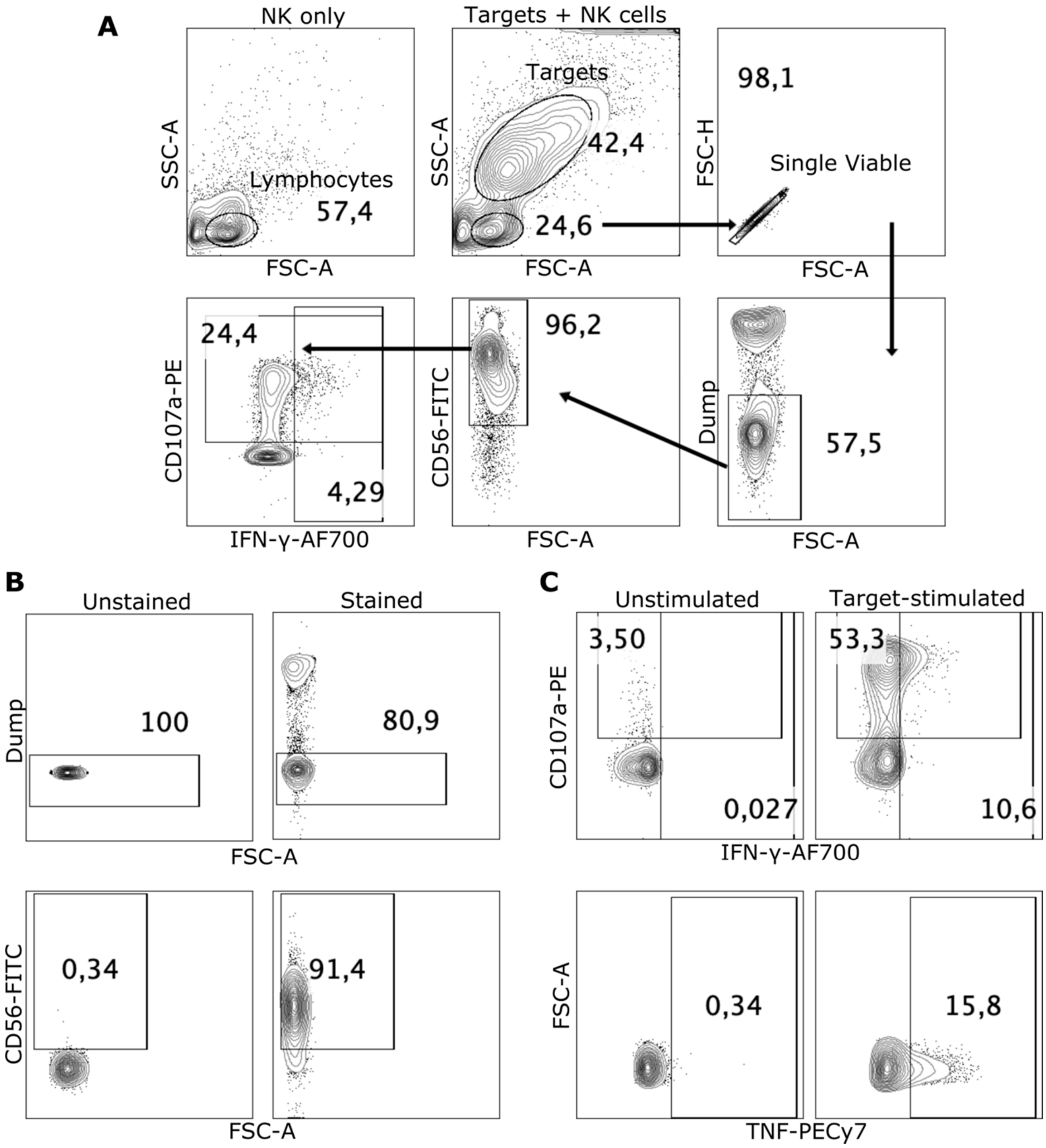

Supplementary Figure S7. Flow Cytometry Plots Demonstrating Gating Strategies for Positive Cell Identification

A) Gating sequence utilized for NK cells co-cultured with target cells: (i) Size-based gating, (ii) removal of doublets, (iii) Viability+CD3APC-Cy7-based gating (Dump), (iv) Identification of NK cells (CD56+ CD3-), and (v) identification of CD107a+ or IFN-γ+ cells. B) Representative flow cytometry scatter plots showing the gating strategy for Fixable viability dye-eflour780 and CD56-FITC staining after gating on lymphocytes (FSC/SSC), along with corresponding unstained controls. C) Representative flow cytometry scatter plots illustrating the gating strategy for CD107a-PE, IFN-γ-AF700, and TNF-PECy7 staining of target-stimulated cells and unstimulated controls.
